# Does off-farm employment contribute to chemical fertilizer reduction? New evidence from the main rice-producing area in Jilin Province, China

**DOI:** 10.1371/journal.pone.0279194

**Published:** 2022-12-16

**Authors:** Yu Lang, Guixia Wang, Sonny Gad Attipoe, Dongxu Han

**Affiliations:** 1 College of Economics and Management, Jilin Agricultural University, Changchun, China; 2 Department of Agriculture, Environmental and Health Science Education, University of Education, Winneba, Ghana; 3 College of Horticulture, Jilin Agricultural University, Changchun, China; Szechenyi Istvan University: Szechenyi Istvan Egyetem, HUNGARY

## Abstract

Frequent transfer of rural labor to cities in developing countries significantly impacts agricultural production. However, whether off-farm employment can promote chemical fertilizer (CF) reduction is still controversial. This study incorporates business scale (BS) and fragmentation degree of arable land (FDAL) into the theoretical analysis framework, shedding light on regulating effects of arable land resource endowment in the process of off-farm employment which influences CF application under different BS and FDAL scenarios. It also empirically tests the theoretical framework by employing the survey data of 318 rice farmers in Jilin Province. The results indicate that: (1) off-farm employment, in general, promotes the adoption behavior of machinery by farmers, and mechanical tillage can significantly reduce the intensity of CF application. (2) If farmers have large BS and non-dispersed farmland parcels, contiguous cultivation will meet the scale threshold for mechanical farming and obtain economies of scale to reduce the intensity of CF application. (3) If farmers have small BS and dispersed farmland parcels, the scale threshold of mechanical farming cannot be met. In order to stabilize agricultural production, farmers will increase the intensity of CF application. According to the empirical results, we put forward some suggestions.

## 1. Introduction

By 2050, the global population will exceed 9 billion, resulting in greater demand for food, cultivated land, water, and other resources. At the same time, it is faced with enormous challenges such as environmental degradation, food safety, and climate change, which are difficult to maintain food’s quantity and quality safety. "Food insecurity" has become one of the most critical and complicated problems in Asia and even the world [1, 2]. Different from modern agriculture with environmental protection and high efficiency in most developed countries, most developing countries still need huge labor input, extensive application of CF, and agricultural environmental pollution in exchange for the increase of grain output. Due to the food insecurity caused by rapid urbanization, unstable food supply, and increasingly serious environmental pollution in recent years, more and more Asian countries have tried to invest in overseas farmland. Although these countries can continue to modernize their own development models and improve food security in this way, overseas food cultivation still needs to comply with the policy management and environmental protection requirements of the local countries, which may lead to higher costs and increase the uncertainties of food security [3]. Asia’s food system varies with the economic structure and food production capacity of Asian countries. There are differences in resource endowments and economic development levels among different countries, and their agricultural development also presents diversified development patterns. However, the same thing is that the agricultural labor force shifts to cities with higher productivity, which weakens the relevance of the agricultural sector [4].

Food system includes agricultural development, environmental sustainability, food supply, food consumption and nutrition access. Therefore, climate change, smallholder sustainability, labor transfer, environmental degradation and non-genetic diseases and their global solutions are all related to the food system [5, 6]. In developed countries, Denmark and South Korea regard cooperatives as farmers’ organizations and the role of cooperatives are limited to crop processing, logistics services and the supply of agricultural materials. As co-owners of cooperatives, farmers will not jointly produce or merge their land to pursue scale production but will cooperate extensively to achieve high production efficiency. Due to the difference in resource endowment, the average farm size in Denmark is far larger than that in South Korea. In Denmark, farmers produce organic agriculture according to cooperatives and export to other countries. However, cooperatives in South Korea have only experienced a short period of development, and the main purpose is to meet domestic demand. A certain amount of CF and pesticides still need to be invested to maintain production and reduce pests and diseases [7]. However, under the background of the continuous transfer of off-farm employment, developed countries have adapted to and formed a relatively stable agricultural production mode, and have completed or gradually changed to green and environmental protection. Denmark can rely on cooperatives to carry out large-scale organic agricultural production, maintain high production efficiency and protect the environment. Similar to China, South Korea has less and scattered arable land and more natural environment unsuitable for agricultural production. However, compared with South Korea, China has a larger population. In order to ensure grain output and improve the productivity of arable land, it can only rely on the massive input of CF, resulting in serious arable land degradation and environmental pollution. A study on Vietnam’s agriculture shows that Vietnam’s agricultural output value accounts for 30% of the national GDP and has become one of the largest agricultural exporters in the world. Although Vietnam is currently facing a shortage of crop purchasing system and advanced financing forms, the construction of public warehousing and developing commodity exchanges will undoubtedly improve the financing environment of Vietnam’s agriculture and increase farmers’ agricultural production and construction funds [8]. Agriculture supports Vietnam’s economic development, but China’s economic growth does not depend on agricultural exports, and agricultural production is focused on ensuring national food security. Like China, India is one of the most populous developing countries and its agricultural production is highly dependent on the application of CF, which is in excess [9], but unlike China, India has abundant arable land, sufficient water and a suitable climate for farming, and the reduction of CF and agrochemicals has greater potential and feasibility. However, in China, where arable land is scattered and insufficient, water resources are scarce, and climate differences are obvious, it is undoubtedly a huge challenge to achieve long-term food self-sufficiency, and the current solution is to invest heavily in CF and pesticides to help increase food production.

With the acceleration of global urbanization and industrialization, most developing countries are experiencing two phenomena: population urbanization and rural hollowing out [10–14]. China is no exception. Affected by the rising opportunity costs of agricultural production, a large outflow of rural labor is gradually being attracted to off-farm employment [15–18]. According to statistics, 85% of rural households have at least one member employed in an off-farm sector [19], and the number of migrant workers in China increased from 145 million in 2009 to 290 million in 2019 [20, 21]. The redistribution of labor force between urban and rural areas constantly impacts the traditional mode of agricultural production, which has a significant impact on household livelihoods and agricultural production [22–25]. Equally remarkable is the tremendous success of intensive agriculture in addressing China’s food security problems since the Green Revolution in the 1950s [19, 26]. China feeds 20% of the world’s population on only 9% of the world’s arable land, but this success results from applying 30% of the global agrochemical [27]. The average amount of CF application in China is 3.6 times greater than the world average [26], and the application intensity of CF is 1.61 times upper the limit of international safety standard (225 kg/ha) [28]. In 2019, the average application of CF in China was 54.035 million tons. An approximately 40% of it was lost to the environment [29], causing serious environmental pollution. Currently, excessive application and inefficient utilization of CF are common in China [30]. Under the current background of urbanization and industrialization, the continuous migration of rural population to cities leads to the reduction of agricultural labor force, and changes in household income and inputs of agricultural production factors.

Some scholars have focused on the impact of off-farm employment on agricultural chemicals investment. As two basic factors in agricultural production, off-farm employment will affect CF application [11]. Therefore, clarifying the feedback mechanism between off-farm employment and CF application is essential to ensuring food security [31], ecological safety [32], and the sustainable use of arable land [33]. Some studies point out that in the context of increasing off-farm employment, farmers’ primary consideration in CF input decisions is cost-benefit analysis, and there are insufficient incentives for CF use in terms of returns for subsistence farmers [34]. When the opportunity cost of agricultural production is high, farm households rely mainly on off-farm employment for their livelihood and are unable to meet the labor needs of agricultural production. This has led to a decrease in the intensity of land production factor inputs, changes in the type of use, and even parallel with the abandonment of land [11, 35], which has a direct negative impact on CF inputs. Shi et al. [36] showed that off-farm employment has little income effect but leads to a large labor loss effect, as evidenced by the shift from double-season to single-season rice cultivation. The non-intensive tendency of rice production indicates that the increase in off-farm employment reduces CF inputs. Zhang et al. [37] studied the influence of returning to the countryside after participating in off-farm employment on CF application. After solving the problems of self-selection bias and endogeneity, the results show that having the experience of urban-rural migration is helpful to reduce the amount of CF input in rice production. Chang and Mishra [38] used a quantile regression model to analyze the impact of off-farm employment on the intensity of CF input. The results showed that off-farm employment would reduce the input of CF, and it was more obvious when the quantile was higher. At this point, the intensity of CF application decreases, as the supply of off-farm labor increases. However, it has also been suggested that rural labor migration has a factor substitution effect on CF application; that is, off-farm employment promotes CF inputs [39–41]. Since the urban and rural household registration system was liberalized in the 1980s, off-farm employment has become an important strategic option, allowing farm households to improve their household livelihoods [42]. Off-farm employment can compensate for agricultural expenditures by providing sufficient remittance effects [43] and promoting the redistribution of agricultural production factors. Under the constraints of labor cost and budget, farmers apply additional CF using remittance inflows to compensate for labor loss while maintaining agricultural output [40, 41, 44]. Based on the perspective of farmers’ risk preference, the research shows that risk-averse farmers use off-farm employment to balance consumption, which leads to an increase in CF application [45]. Ma et al. [46] pointed out that only 15% of apple growers participated in off-farm employment, and the results after controlling the deviation by the treatment effects model showed that off-farm employment had a positive and significant impact on the application of CF and pesticides. In addition, the outflow of young and middle-aged labor force leads to a decline in the quality of peasants. Agricultural production has changed from intensive cultivation to extensive management. Meanwhile, CF application has also changed from multiple fertilization to one-time fertilization. The low awareness of ecological protection among the elderly and women with low literacy levels in villages is exacerbating the difficulty of controlling agricultural non-point source pollution.

However, other scholars believe that whether off-farm employment can promote CF reduction depends on the situation. Mathenge and Smale [47] estimated off-farm income, as well as off-farm income, income from labor on other farms, and all off-farm income combined on CF inputs for three crops: major food crops (maize), emerging cash crops (vegetables), and traditional export crops (coffee/tea). The results showed that under certain conditions, off-farm earnings had a negative effect on CF inputs for both major food crops and emerging cash crops in Kenya, but the effects on the CF inputs of traditional cash crops are weak but positive. A study based on the data of 2419 farms in England and Wales holds that off-farm employment has a significant impact on agricultural production. With the increase in off-farm working hours, the input intensity of factors clearly related to environmental damage is different. Specifically, when off-farm employment is between 430–900 hours, environmental protection factor inputs will increase under the influence of off-farm employment, while CF input intensity will decrease with the increase in off-farm employment. Total input intensity in agriculture will increase when off-farm hours of work are within 200–430 hours and will decrease when off-farm employment hours of work exceed 430 hours [48]. The study by Zhang et al. [11] interestingly reveals the difference in the effect of off-farm employment on CF inputs in mountainous areas and plains. In particular, while off-farm employment and CF inputs showed an inverted U-shaped relationship in mountainous areas, a significant positive relationship was observed in the plains, where with the increase in off-farm employment, scale effects were generated in the plains through large-scale land management, thus reducing the intensity of CF application. Studies on the relationship between off-farm employment and CF inputs have attracted the attention of many scholars and provided some insights. However, there is no consensus among established studies as to whether off-farm employment can promote CF reduction.

All of the above studies suggest that whether off-farm employment affects CF application may be influenced by differences in the duration of off-farm industry, different crops grown, and differences in sources of income earned from engaging in different off-farm activities, resulting in different outcomes. This paper holds that the reason for the disagreement is that existing studies have mainly followed the single analysis logic of "off-farm employment—factor substitution—CF application", which leads to the simplification of factor allocation caused by off-farm employment, and thus ignores the differences in factor allocation and the potential of CF reduction under the conditions of different arable land resource endowment. The optimal factor selection behavior differs under different arable land resource endowment scenarios. A larger BS and lower FDAL cannot overcome the disadvantages of small plot size, therefore farmers usually use CF instead of labor, which increases the intensity of CF application. On the contrary, the economies of scale generated by contiguous cultivation induce long-term investment behavior of farmers, resulting in CF reduction effects. It can be inferred that the reallocation of production factors due to off-farm employment in different arable land resource endowment scenarios may lead to an obvious contrast in CF application.

Therefore, the contribution of this paper mainly has two points: firstly, we discuss the regulatory role of arable land resource endowment in the process of off-farm employment affecting CF application. Although studies have explored the regional heterogeneity of the impact of off-farm employment on carbon fiber application intensity under different terrain conditions, they have not revealed the reasons for the differences in farmers’ fertilizer application behavior under the same geographical environment. In fact, as the main body of CF application, farmers with different arable land resource endowments have different fertilization behaviors. However, the existing research has not been paid attention to.

Secondly, we further analyzed the intermediary role of mechanical farming in the impact of off-farm employment on CF application and revealed the internal mechanism of off-farm employment leading to the difference in CF application.

This paper reveals the relationship among off-farm employment, factor allocation, and CF reduction under different arable land resource endowment scenarios, so as to breaking through the one-way thinking that off-farm employment affects CF input and explaining the internal mechanism of off-farm employment that leads to different allocations of production factors that affect CF application. In general, this paper is divided into seven parts. Following the introduction, the second part is literature review. the third part proposes a research hypothesis based on the theoretical analysis, the fourth part introduces the research area and data sources, and describes the econometric methods, the fifth part analyzes the empirical results, the sixth part provides a discussion, the seventh part draws a conclusion and policy implication.

## 2. Literature review

Off-farm employment plays an important role in the livelihood of most households in developing countries. Family members engaged in agricultural production are not constrained by any time or place and earn off-farm remuneration by participating in productive activities other than agricultural production, thereby alleviating household mobility constraints. At present, many studies have made important contributions to understanding the reasons [49–53] and results [54–56] of farmers’ participation in off-farm employment.

This paper argues that off-farm employment brings about both a household labor shortage and an easing of liquidity constraints. In the context of the increasing prevalence of off-farm employment, in order to maintain agricultural production as much as possible, farm households tend to invest their off-farm income in agriculture to compensate for the impact of labor shortage. Thus, how farmers choose factors to replace labor shortage is the focus of this paper. In contrast, off-farm work is able to transform the labor factors with which agricultural production competes into other capital factors and use them in agricultural production as a way to mitigate the labor loss effect and maintain household livelihoods.

The literature on the role and impact of off-farm employment is divided into two main parts. The first part is the impact of off-farm employment on agricultural production. For example, Lisa Pfeiffer et al. [57] studied the impact of off-farm employment on agricultural production based on data from the Mexican Rural Household Survey and found, after analysis, that off-farm employment has a negative impact on both agricultural output and agricultural labor use. Another study based on data from a sample of fruit growers in Shandong Province similarly showed that off-farm employment reduced the likelihood and intensity of fruit production [58]. However, the results of Raphael O. Babatunde [59] challenge the above analysis: in addition to the fact that the marginal effects of off-farm employment and income earned from agricultural production are the same for household consumption, the case based on Kwara State suggests that an increase in off-farm employment can instead boost highly intensive agricultural production and increase food production. Adem Endiris et al. [60] similarly showed that participation in off-farm activities increases the average probability of household food security, that households not involved in off-farm employment have a lower average probability of food security, and that off-farm employment significantly increases the likelihood of household food security. A study of Norwegian farm data from 1991–2005 concluded that the impact of off-farm employment on agricultural production tends to increase and then decrease with the duration of off-farm employment and does not systematically affect farm technical efficiency [61]. Xin Deng et al. [62] studied how off-farm employment affects land abandonment. The research shows that before the off-farm employment rate reaches 46%, the land abandonment will be positively affected, while when the off-farm employment rate exceeds 46%, the abandonment of farmland will decrease, and there is a positive correlation between the off-farm employment and the abandoned area of farmland. Subsequently, DENG Xin et al. [63] studied the effect of off-farm employment on land rental. Specifically, off-farm employment indirectly influenced the spatial clustering of the land rental, and the two showed an inverted U-shaped curve relationship with a turning point of 55.55% off-farm employment; off-farm employment and the land rental area also showed an inverted U-shaped relationship with a turning point of 56.22% off-farm employment.

The second part of the literature focuses on the impact of off-farm employment on changes in the allocation of factors of production in agriculture. Off-farm employment relaxes the liquidity constraints on household agricultural production and seeks to increase the purchase of other production alternatives to household labor, leading to an increase in other factors of agricultural production inputs [57]. Wanglin Ma et al. [64] noted that off-farm employment positively and significantly contributed to farm households’ farm machinery use behavior, but had a significant and negative impact on agrochemical input costs. In particular, additional use of farm machinery significantly increased maize yield by 74 kg/mu and agrochemical costs by 99 Yuan/mu, but participation in off-farm employment would reduce agrochemical costs by 47 Yuan/mu. Another study found that off-farm employment was not significantly related to land leveling investment, but negatively affected agricultural water consumption, while the percentage of student mobility in the household had a significant positive effect on land leveling investment [65]. Wanglin Ma et al. [46] used the treatment effects model to address the sample use bias and noted that the effects of off-farm work and agricultural production were consistent in terms of income and that there was a significant and positive marginal effect between increased off-farm work and both CF and pesticide inputs. Existing studies have laid the foundation and ideas for this study, and the present study contributes to the literature by analyzing the effect of participation in off-farm employment on the intensity of CF.

## 3. Theoretical perspective and research hypothesis

The new economics of labor mobility (NELM) [66, 67] theory articulates a view: External work and participation in off-farm employment are rational choices made by farm households based on the household livelihood level, aiming to diversify revenue sources. Off-farm employment can make up for the loss of agricultural production (labor shortage effect) caused by the loss of human capital of rural families by improving the level of family income (remittance effect) [68], extending the boundaries of income constraints among those rural members left behind. External employment also promotes household purchases of more capital-intensive, labor-saving agricultural production technologies and services to maintain land productivity [69]. This view is reflected in the input of agricultural production factors such as the increased purchase of capital, technology, and services by farm households to reallocate factors of production. Since farmers have different resource endowments of arable land, different forms of factor allocation lead to different CF reduction performances.

### 3.1 Off-farm employment and allocation of agricultural production factor

Rural land in China is collectively owned and is characterized by two main features: small scale and high fragmentation. As a result, clarifying land property rights, improving the land exchange market, and promoting the centralized circulation of farmland contract management rights are considered critical measures if agricultural production is aiming to gain economies of scale [70, 71]. In recent years, farmers with off-farm employment capacity allocate their labor force to work activities, thus accelerating the transfer of the possession of land contract management right to large-scale households with advantages in agricultural production. The ratio of farmers cultivating their own contracted land versus transferable land has shifted from 97:3 in 1996 to 81:19 in 2008 [72]. However, in more than 86 million hectares of contracted land, only approximately 25 million hectares have been transferred, and the remaining 61 million hectares of land have not yet been transferred, with a transfer rate of only 29%. Although there is a trend toward centralization in the scale of land management in China, it remains small and highly fragmented [73]. Currently, China’s arable land shows a wide disparity in the scale of business and significant differences in the number of plots.

The existence of factor allocation effect in agriculture leads to a significant substitution relationship between material factor input and labor force. The increase of off-farm employment leads to a decline in the quality of labor force and an increase in the relative price of agricultural labor force. To avoid losses in agricultural output and efficiency, farmers adjust the structure of factor inputs in response to changes in the relative price of factors. On the one hand, the productive service outsourcing organizations have induced a strong demand for machinery services to replace labor force, on the other hand, the low price of CF promotes the substitution of CF factors for labor factors. Limited by the high investment threshold and asset specificity of agricultural machinery and reduction technologies, farmers generally have little incentive to invest in the short term [71]. Outsourcing services of agricultural production turn technological progress into an endogenous driving force to promote the spread of agricultural machinery. Changes in the input structure of agricultural production factors not only depend on relative prices, but are also influenced by the resource endowment of arable land, and the status of land transfer is a basic prerequisite for factor allocation in the context of off-farm employment. The use of agricultural machinery requires a certain spatial range for reciprocal cyclic movement, but the small BS and scattered plot locations increase the difficulty of replacing labor with machinery. In contrast, artificial fertilization is often carried out in small BS and fragmented land, and the contiguous BS is prone to cause the confusion of artificial fertilization order and high labor costs.

### 3.2 Changes in agricultural production factors and CF application

**Fig 1:**
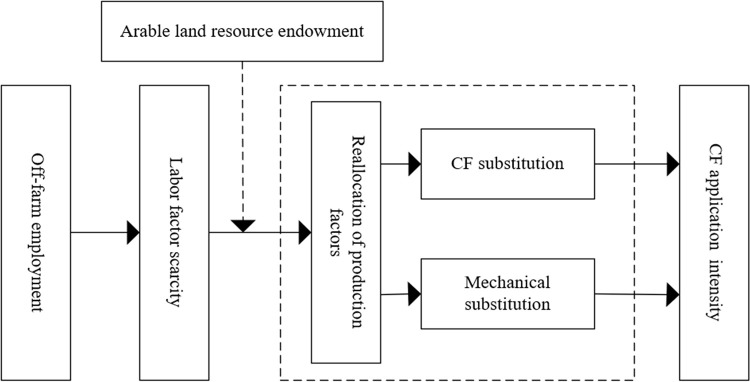
Theoretical framework analysis. Source: The authors’ compilation.

The land transfer caused by off-farm employment does not directly act on CF inputs, but provides the basis and prerequisite for the adjustment of the factor structure. When the remittance effect caused by off-farm employment is greater than the labor shortage effect, that is, the income from engaging in off-farm activities is larger than the loss from abandoning agricultural production, farmers tend to retain part-time status and seek to reallocate production factors to stabilize agricultural production. In discerning with the relationship between off-farm employment and CF inputs under different arable land resource endowments, two main types of scenarios can be summarized. First, the BS is small and the FDAL is high. Smaller plot scales cannot reach the scale threshold for mechanical intervention, so CF is a better substitute for labor shortages than machinery, and farmers tend to apply more CF. Second, the BS is large and the FDAL is low. With the continuous increase of labor and leisure costs, the substitution of CF for labor shortage will lead to higher production costs under a large plot scale. Meanwhile, the larger plot scale provides conditions for continuous planting and market capacity for socialized service outsourcing. Therefore, machinery is easy to intervene in agricultural production and produces the scale effect of CF reduction. At this time, farmers tend to get involved in the division of labor system by purchasing contiguous mechanical farming services. CF reduction depends not only on Farmers’ awareness of green production but also on the adoption of CF application machinery with the progress of agricultural reduction technology. Not only do the development of agricultural machinery and the progress of CF application technology improve labor productivity, but also reduce the intensity of CF application. The CF reduction effect of mechanical fertilization is mainly reflected in two aspects: The first is the round-tripping investment effect of reduction machinery. Farmers can effectively overcome the high investment threshold and strong asset specificity of mechanical equipment by purchasing mechanized deep fertilization and controlled release service. The second is the roundabout introduction effect of reduction technologies. By purchasing technology-intensive products and services, farmers apply fertilizer reduction technology directly to agricultural production [74], which leads to a huge potential for CF reduction. However, as long as agricultural machinery is involved in agricultural production, it is necessary to meet the requirements of mechanical farming in terms of plot leveling and area concentration. Fig 1 shows the theoretical framework analysis of this study. Based on the above analysis, the following hypotheses are proposed in this paper.

H1: Off-farm employment in general can reduce the intensity of CF application through mechanical substitution.

H2: If farmers have a small BS and dispersed farmland parcels, off-farm employment will increase the intensity of CF application.

H3: If farmers have a large BS and non-dispersed farmland parcels, Off-farm employment will increase the machinery use behavior of farmers, which in turn reduces the intensity of CF application.

## 4. Materials and methods

### 4.1 Study area and data sources

The study area of this paper is Jilin Province, which is located in the middle of Northeast China and is one of the three cold black soil belts in the world [75]. There are plains, valleys, mountains, and hills that can be cultivated in Jilin Province, making this area the main production base with soft and fertile soil that is suitable for the cultivation of high-quality japonica rice in China. The province has a perennial rice cultivation area of 800,000 hectares, which is planted in the middle, east, and west, mainly in the Tumen River, Yalu River, Songhua River, Neng River and Liao River basins. As China’s main grain-producing region and the "ballast" of China’s food security, Jilin Province has produced more than 70 billion pounds of grain for eight consecutive years with the contribution of black land exceeding 80%. However, for decades, predatory development and the heavy use of nutrients have led to a long-term excess of CF inputs. Agricultural surface pollution and soil consolidation problems are becoming increasingly serious, the ecological environment continues to deteriorate, the black soil layer is thinning, and the soil organic matter content is declining rapidly [76, 77]. In addition, with the increasing level of urbanization, the rural population of Jilin Province has been flowing out so it has decreased from 12.9 million in 2005 to 11.23 million in 2019. During these 15 years, urban population increased from 14.26 million to 15.68 million and rural hollowing rate had been accelerating [78]. Therefore, Jilin Province is a good representative for investigating how off-farm employment affects CF inputs. The selection of Jilin Province as the study area reflects the considerable importance of controlling agricultural non-point source pollution, contributing to protecting black land, and even ensuring national food security.

The study applied a multi-stage sampling technique to select respondents among rice farmers. Interviews were conducted in 11 county-level cities, counties and districts and 22 villages and towns under six prefecture-level cities in Jilin Province, namely, Baicheng City (Taonan City), Changchun City (Jiutai District, Gongzhuling City), Jilin City (Yongji County, Shulan City), Tonghua City (Mehekou City, Tonghua County), Yanbian City (Yanji City, Longjing City), and Songyuan City (Changling County, Qianguo County) from September to November 2020 (each county-level city, county, and district selected 2 villages and towns) for a total of 331 questionnaire-based interviews with rice farmers (Table 1). These areas were selected because they are typical rice-growing areas. We had local contacts such as village officials, fertilization technicians and farmers in the area to ensure that we could reach informants. They hope to obtain practical policy recommendations from our work to control agricultural non-point source pollution and promote CF reduction as well as enhancing CF efficiency. Before conducting the survey, the research team conducted a two-day training for the investigators to ensure that the questions had been asked in a standard way so that they could get the general situation and the overall fertilization information of the village from local acquaintances. Because our core purpose was to capture the influences on farmers’ CF application behavior, we added personal characteristics, family structure, farmland management, CF purchase and application behavior, green technology awareness, and attitudes towards agricultural non-point source pollution and ecological compensation in the design of the questionnaire. During the in-depth household interviews, we avoided using terms such as "over-fertilization" because farmers were not aware of or sensitive to their over-fertilization. During the communication process, we only asked questions and filled in results according to the farmers’ answers, rather than providing farmers with answers so as to avoid guiding their answers and then affecting the accuracy of the research data. This paper used a combination of semi-structured in-depth interviews and questionnaires to collect multiple dimensions of information, and in-depth interviews based on questionnaire content could better help us understand complex behaviors, experiences, and perspectives deeply. Each family interview lasted approximately 30 minutes. After that, rewards were given to the interviewees who were recruited according to the snowball principle. Moreover, additional rewards were given to those who had introduced these interviewees to us. In the investigation, we found that the fertilization types of farmers are mainly nitrogen, phosphorus, and potassium fertilizer, compound fertilizer and formula fertilization by soil testing. Since the fertilization habits, order and dosage of different farmers vary greatly, we used the CF application rate per unit area of sample farmers to express the CF application situation for the convenience of statistics. During this investigation, the research team issued a total of 331 questionnaires. After collection and sorting, 13 questionnaires with missing or invalid data were eliminated and 318 valid questionnaires were collected for an effective rate of 96.1%.

**Table 1:** Overview of the sources of survey data.

Research area (prefecture-level city)	Research area (county-level cities, counties, districts)	Survey of villages and towns	Distribution of questionnaires (copies)	Valid questionnaires (copies)	Efficient(%)
Baicheng City	Taonan City	Happy Village	15	14	93.33
		Chunhua Village	11	11	100
Changchun City	Jiutai District	Qiangang Village	13	11	84.62
		Hongguang Village	18	17	94.44
	Gongzhuling City	Baya Town	22	20	90.90
		Nanwaizi Village	19	19	100
Jilin City	Yongji County	Hejia Village	18	16	88.89
		Lugiazi Village	19	19	100
	Shulan City	Fanshen Village	12	11	91.67
		Shengli Village	18	18	93.8
Tonghua City	Mehekou City	Hailong Town	15	15	100
		Xinhe Village	9	7	77.78
	Tonghua County	Tongxin Village	18	18	100
		Fusheng Village	20	20	100
Yanbian Prefecture	Yanji City	Pinggang Village	11	11	100
		Badao Village	14	13	92.96
	Longjing City	Xinhua Village	10	10	100
		Dongsheng Village	18	18	100
Songwon City	Changling County	Dongxing Village	15	15	100
		Thirty-two Villages	11	11	100
	Qianguo County	Hongguang Farm	12	11	91.67
		Qianyingzi Village	13	13	100
Total	11	22	331	318	96.1

Source: The authors’ compilation based on the research groups field research (2020).

### 4.2 Ethical considerations

During the field investigation, all ethical issues were properly addressed. First, The Jilin Agricultural University Graduate Research Ethics Committee in China authorized this investigation. Secondly, in the preparation stage of the interview, we first obtained the consent authorization from the Zone Executive Officer (ZEO). On this basis, the exact information in the consent form was communicated to each target respondent by phone or SMS, and their verbal consent was obtained. Subsequently, during the field research, we invited the target respondents to fill out an informed consent form to certify their consent to the survey. All interviewers were informed that they could withdraw from the survey activities at any time. Our research activities do not involve minors. In addition, the research group is composed of graduate students, teachers and other researchers, and does not belong to government organizations and foreign-related surveys, so it does not need the approval of statistical departments.

### 4.3 Model setup and variable selection

How off-farm employment, BS, and FDAL affect CF application is the focus of this paper, which explores the differences in the intensity of CF application affected by off-farm employment under different arable resource endowment scenarios. This paper draws on the modeling approach of Liang et al. [71] to construct econometric models without and with interaction terms, as well as farmers’ machinery adoption behavior, respectively. Where the formula without the interaction term is constructed as follows:

$$\ln(Cfertilizer_{i})=\beta_{0}+\beta_{1}Naemploy_{i}+\beta_{2}Bscale_{i}+\beta_{3}Nplots_{i}+\sum_{j=1}\beta_{4j}X_{i}+D_{i}+\varepsilon_{i}$$ (1)

$$\ln(Cfertilizer_{i})=\beta_{0}+\beta_{1}Bscale_{i}+\beta_{2}Nplots_{i}+\beta_{3}{\mathrm{Mac}}_{i}+\sum_{j=1}\beta_{4j}X_{i}+D_{i}+\varepsilon_{i}$$ (2)

$$Mac_{i}=\beta_{0}+\beta_{1}Naemploy_{i}+\beta_{2}Bscale_{i}+\beta_{3}Nplots_{i}+\sum_{j=1}\beta_{4j}X_{i}+D_{i}+\varepsilon_{i}$$ (3)

The variables in Formulas (1), (2) and (3) are selected as follows:

- The predicted variable. Cfertilizer_i_ represents the intensity of CF application by farm households, measured by the average application amount of CF per unit of farmland (ha) by farm household i. Mac_i_ represents the use of machinery by farm households, measured by whether farmer i used machinery in fertilization.
- The explanatory variable. Naemploy_i_ stands for off-farm employment, which is expressed by the proportion of off-farm income of farmer i in total household income. Bscale_i_ represents the BS of farmer i, which is reflected by the total scale of rice planting. Nplots_i_ represents the FDAL of farmer i, which is reflected by the number of farmland plots.
- The control variables. X_i_ is a control variable that includes personal characteristics (gender, age, educational level, health condition), household characteristics (total family population, number of labor force, household social capital), production and business characteristics (years of rice production, production organization business model, soil fertility), and external environment characteristics (technical training, livestock breeding, access to information) of agricultural decision-makers. During the investigation, we found that three cooperation modes may generate in the mode of production organization and operation, like "family self-employment", "Cooperation between enterprises and farmers" and "Cooperation between cooperatives and farmers". Among them, "family self-employment" is manifested as self-management of agricultural production by farmers, self-production, and self-sold. "Cooperation between enterprises and farmers" is an economic contract signed between enterprises and farmers to carry out integrated management, but farmers are more restricted by enterprises and have unequal market status. Under this model, farmers transfer land to enterprises. Receiving regular salary payments from the enterprise that takes planting income, farmers are not liable for operating costs. Unlike the first two, "Cooperation between cooperatives and farmers" is voluntarily united by the same kind of agricultural products operators and sets up a democratic management of mutual economic organizations. Having no constraints and greater rights, farmers take the production income in the mode of cooperative unified management and production and operation.
- The dummy variables. D_i_ is a dummy variable to control for regional fixed effects such as climate, pests and diseases; *ε*_*i*_ is the stochastic disturbance team; β_0_ is the intercept term; and β_1_、β_2_、β_3_、β_4_、β_4j_、β_5j_ are the parameters to be estimated. In this study, the predicted variable Cfertilizer_i_ has to be processed with logarithm to weaken the potential heteroscedasticity of the model [79].

The formula containing the interaction terms is constructed as follows:

$$\ln(Cfertilizer_{i})=\beta_{0}+\beta_{1}Naemploy_{i}+\beta_{2}Bscale_{i}+\beta_{3}Naemploy_{i}\times Bscale_{i}+\beta_{4}Nplots_{i}+\sum_{j=1}\beta_{5j}X_{i}+\varepsilon_{i}$$ (4)

$$\ln(Cfertilizer_{i})=\beta_{0}+\beta_{1}Naemploy_{i}+\beta_{2}Nplots_{i}+\beta_{3}Naemploy_{i}\times Nplots_{i}+\beta_{4}Bscale_{i}+\sum_{j=1}\beta_{5j}X_{i}+\varepsilon_{i}$$ (5)

Naemploy_i_×Bscale_i_ and Naemploy_i_×Nplots_i_ respectively represent the interaction term between off-farm employment and BS, and the interaction term between off-farm employment and FDAL. In this paper, the interaction terms are centralized to overcome potential multicollinearity.

Endogeneity is a common problem in studying farmers’ behavioral decisions and the factor that influences them [80]. It is mainly reflected in two aspects: the first is the self-selective bias. Farmers’ CF application behavior is influenced by observable factors (e.g., gender, age, etc.) as well as immeasurable factors (e.g., individual physical ability). For example, the more physically able an individual farmer is, the more likely he or she is to forego hiring machinery, thereby reducing the cost of hiring machinery. This may lead to overestimation of the effect of off-farm employment on CF application reduction by farm households when conducting OLS estimations. The second is the simultaneity bias (reverse causality). This is a special kind of omitted variable problem. Farmers may have a significant reduction in CF input costs and demand for labor as a result of mastering new CF reduction technologies, and then their household members may be more inclined to engage in off-farm employment, thereby increasing their household income levels. Therefore, selecting the appropriate instrumental variables is one of the most effective ways to address endogeneity.

In the literature related to the study of urban and rural labor transfer employment, migration networks are often used as an instrumental variable to examine individual or household participation in out-of-home employment [81, 82]. Rural China is a typical human society, with family tying as the core bond and a network of acquaintances relying on neighborhood relationships. People obtain employment information, referrals and help from acquaintance networks built up by villagers who are migrating for employment to reduce the migration cost and increase the possibility of going out to participate in off-farm employment [83]. Local off-farm employment networks influence farm households’ off-farm employment behavior through acquaintances and off-farm economic development. Accordingly, this paper selects the village-level off-farm employment network as an instrumental variable for off-farm employment, measured by the village-level off-farm employment rate. The topography of Jilin Province is high in the east and low in the west, divided into 3 parts with the western foot of Dahei Mountain as the boundary. The eastern part is the main part of the Changbai Mountain Range, the central part is mostly low hills, and the western part is the pre-mountain terrace and the Songliao Plain. Farmers’ arable land conditions are naturally segmented by topography, which determines the BS and FDAL, and subsequently affects farmers’ CF application behavior. Accordingly, the main terrain type for the farming land was selected as an instrumental variable for the BS and the FDAL.

### 4.4 Descriptive analysis of variables

The meanings of the variables and a descriptive statistical analysis are shown in Table 2. The number of sample farm households involved in off-farm employment was 249, accounting for 78.3% of the overall sample. On average, the average CF application per unit of arable land area (ha) for the overall sample of farmers in Jilin Province was 917.4 kg, The average CF application per hectare was higher for sample farmers not participating in the off-farm employment group (938.48 kg) than for those who participated in off-farm employment (911.57 kg). The average BS of the sample farmers was 6.33 hectares, and the average number of farmland plots was 6.59. The average BS in terms of land for farmers involved in off-farm employment (6.47 ha) was larger than that of farmers not involved in off-farm employment (5.80 ha), while the FDAL of farmers not involved in off-farm employment (7.43 plots) was higher than that of farmers involved in off-farm employment (6.36 plots). The mean value of machinery adoption was significantly higher for the sample farmers involved in off-farm employment (0.61) than for the sample farmers not involved in off-farm employment (0.30). The above analysis shows that there are general laws on the impact of participation in off-farm employment, BS, FDAL and the adoption of machinery on the intensity of CF application.

**Table 2:** Variable meanings and descriptive statistics.

Variable Name	Variable Meaning and Assignment	Mean and standard deviation of variables			Difference in Means
		Overall	Not engaged in off-farm employment	Engaged in off-farm employment	
		(n = 318)	(n = 69)	(n = 249)	
Cfertilizer	CF application per hectare (kg)	917.41 (143.90)	938.48 (137.44)	911.57 (145.09)	26.91
Bscale	Total rice planting scale (ha)	6.33 (3.90)	5.80 (3.47)	6.47 (4.00)	-0.67
Nplots	Number of plots of arable land (blocks)	6.59 (5.59)	7.43 (6.89)	6.36 (5.14)	1.07
Mac	Use mechanical fertilization (yes = 1, otherwise 0)	0.54 (0.50)	0.30 (0.46)	0.61 (0.49)	-0.31***
Gender	Gender of farmers (male = 1, otherwise 0)	0.65 (0.48)	0.7 (0.46)	0.63 (0.48)	0.07
Age	Age of farmers	58.5 (8.67)	58.33 (8.43)	58.56 (8.72)	-0.23
Education	Years of education of farmers	5.46 (1.76)	5.43 (1.61)	5.46 (1.80)	-0.03
Health	Health condition of farmers Poor = 1, fair = 2, healthy = 3	2.42 (0.67)	2.46 (0.65)	2.41 (0.68)	0.05
population	Number of family members (persons)	4.07 (1.37)	2.65 (1.02)	4.46 (1.19)	-1.81***
labor force	Number of household laborers (persons)	3.44 (1.12)	2.10 (0.52)	3.82 (0.95)	-1.72***
capital	Village officials in the family (Yes = 1, otherwise 0)	0.15 (0.36)	0.01 (0.12)	0.19 (0.39)	-0.18***
production	Years of rice production	35.6 (10.29)	35.97 (10.48)	35.49 (10.24)	0.48
Production organization business model	family self-employment = 1, Cooperation between enterprises and farmers = 2, Cooperation between cooperatives and farmers = 3	1.96 (0.82)	1.91 (0.79)	1.98 (0.82)	-0.07
Sfertility	Soil fertility (Poor = 1, fair = 2, good = 3)	2.14 (0.70)	2.25 (0.69)	2.11 (0.70)	0.14
Technical training	Participate in technical training (Yes = 1, otherwise 0)	0.64 (0.48)	0.64 (0.48)	0.64 (0.48)	0
Livestock breeding	Yes = 1, otherwise 0	0.48 (0.50)	0.54 (0.50)	0.46 (0.50)	0.08
Access to information	By experience = 1, farmer exchange = 2, village committee and market promotion = 3, TV, radio, internet = 4	2.76 (1.19)	2.90 (1.35)	2.73 (1.14)	0.17
Village level off-farm Employment Network(VOEN)	Off-farm employment in villages/Total employment (%)	0.28 (0.11)	0.18 (0.07)	0.30 (0.10)	-0.12***
Type	The main topographic types of arable land (Mountain = 1, Low mountains and hills = 2, Piedmont platform and plain = 3)	2.10 (0.78)	2.09 (0.79)	2.11 (0.78)	-0.02
Regional dummy variables	Set regional dummy variables with county-level cities, counties and districts	-	-	-	-

Note: Standard deviations are in parentheses; *, **, and *** indicate significant differences at the 10%, 5%, and 1% statistical levels for the mean variables of the not engaged in off-farm employment group minus the mean variables of the engaged in off-farm employment group, respectively.

Sources: The author calculating and compilating based on field research.

## 5. Empirical results

### 5.1 Model estimation results

Table 3 shows the results of the CF application intensity model estimation without the inclusion of interaction terms. Regression (1) and (2) represent the results of uncontrolled and controlled rice businesses, respectively. Regression (3) and (4) introduce the FDAL and further control the BS on the basis of regression (4). The results showed that off-farm employment had a significant negative effect on the average CF application per hectare by farmers before and after controlling for the BS and the FDAL. Off-farm employment has generally reduced the intensity of CF application for rice production.

**Table 3:** Model estimation results of influencing factors of CF application intensity of sample farmers (excluding interactive items).

Variables	The predicted variable: ln(Cfertilizer)					
	Regression (1)	Regression (2)	Regression (3)	Regression (4)	Regression (5)	Regression (6)
Naemploy	-0.250*** (0.044)	-0.106*** (0.029)	-0.085*** (0.027)	-0.177*** (0.038)	-	-
Bscale	-	-0.028*** (0.001)	-0.025*** (0.001)	-	-0.025*** (0.002)	-
Nplots	-	-	0.006*** (0.001)	0.011*** (0.001)	-	0.009*** (0.001)
Mac	-	-	-	-	-0.062*** (0.011)	-0.119*** (0.013)
Gender	0.012 (0.014)	0.009 (0.009)	0.006 (0.009)	0.006 (0.012)	0.009 (0.009)	0.007 (0.011)
Age	0.0004 (0.001)	0.0004 (0.001)	-0.001 (0.001)	-0.002 (0.001)	-0.0002 (0.001)	-0.001 (0.001)
Education	-0.005 (0.004)	-0.006** (0.003)	-0.004* (0.002)	-0.002 (0.004)	-0.006** (0.003)	-0.004 (0.003)
Health	-0.010 (0.010)	-0.005 (0.007)	-0.004 (0.006)	-0.007 (0.009)	-0.007 (0.006)	-0.011 (0.008)
population	0.007 (0.009)	0.002 (0.006)	0.001 (0.005)	0.005 (0.008)	-0.001 (0.006)	-0.002 (0.007)
labor force	0.013 (0.012)	0.004 (0.008)	0.003 (0.007)	0.009 (0.010)	-0.002 (0.007)	0.001 (0.009)
capital	-0.043** (0.022)	-0.019 (0.014)	-0.021 (0.013)	-0.040** (0.019)	-0.026** (0.013)	0.040** (0.016)
production	-0.002* (0.001)	-0.002*** (0.001)	-0.001 (0.001)	0.0001 (0.001)	-0.002** (0.001)	0.0002 (0.001)
Production organization business model	-0.029*** (0.010)	-0.013** (0.007)	-0.014** (0.006)	-0.028*** (0.009)	-0.015** (0.006)	-0.027*** (0.008)
Sfertility	-0.103*** (0.010)	-0.034*** (0.007)	-0.033*** (0.007)	-0.085*** (0.009)	-0.030*** (0.007)	-0.067*** (0.008)
Technical training	-0.009 (0.015)	0.004 (0.010)	0.002 (0.009)	-0.011 (0.013)	-0.001 (0.009)	-0.017 (0.011)
Livestock breeding	-0.025* (0.014)	-0.016* (0.009)	-0.021** (0.008)	-0.032*** (0.012)	-0.018** (0.009)	-0.032*** (0.011)
Access to information	-0.006 (0.007)	-0.007 (0.004)	-0.006 (0.004)	-0.005 (0.006)	-0.005 (0.004)	-0.003 (0.005)
Regional dummy variables	yes	yes	yes	yes	yes	yes
Constant	7.260*** (0.074)	7.189*** (0.048)	7.151*** (0.045)	7.171*** (0.064)	7.211*** (0.047)	7.210*** (0.058)
Observations	318	318	318	318	318	318
R^2^	0.453	0.770	0.805	0.594	0.782	0.664

Note: Standard errors are in parentheses

*, **, and *** indicate significance at the 10%, 5%, and 1% statistical levels, respectively.

Sources: The author compilating based on empirical results.

Regression (2) and (3) show that there was a significant negative effect of BS on CF application intensity both before and after controlling for the FDAL. This suggests that the expansion of rice BS reduces the intensity of CF application. When the FDAL is certain, the larger the scale of business, the more likely it is to meet the scale effect threshold of rice production, which makes the reduction of labor factors increase the marginal output rate of mechanical input and reduce the CF application intensity. On the contrary, the smaller the BS, the more the farmers tend to apply more CF to replace the labor factor and increase the intensity of CF application.

Regression (3) and (4) indicate that the FDAL positively affects the intensity of CF application before and after controlling for the BS. This shows that the deepening of the FDAL will lead to an increase in the application of CF. When farmers business at a certain scale, a higher FDAL means that farmers may not be able to capture the scale effect of rice planting, which in turn induces the substitution effect of reduced labor factors to take effect. Therefore, when the rural labor factor decreases, the diseconomy of plot scale will directly lead to farmers’ preference to apply CF instead of labor, thus increasing the intensity of CF application. While the lower the FDAL, it may meet the scale effect threshold and lead to farmers’ demand for mechanical farming, and reduce the intensity of CF application. Regression (5) and (6) represent the regression results of machinery use affecting CF application intensity after controlling the BS and FDAL, respectively. The results indicate that arable land resource endowment can influence CF application intensity by affecting machinery use.

Table 4 reports the model estimation results of farmers’ machinery use behavior model without interaction terms. The results show that off-farm employment has a significant positive impact on the use of agricultural machinery before and after controlling the BS and the FDAL. This is because the Northeast region is vast and sparsely populated. When labor factors are reduced, farmers tend to use machinery as a substitute. As a result, the increase in off-farm employment has generally promoted the use of agricultural machinery.

**Table 4:** Model estimation results of mechanical use behavior of sample farmers.

Variables	The predicted variable: Mac			
	Regression (7)	Regression (8)	Regression (9)	Regression (10)
Naemploy	1.129*** (0.153)	0.819*** (0.139)	0.976*** (0.148)	0.777*** (0.138)
Bscale	-	0.061*** (0.007)	-	0.054*** (0.007)
Nplots	-	-	-0.024*** (0.004)	-0.012*** (0.004)
The control variables	yes	yes	yes	yes
Regional dummy variables	yes	yes	yes	yes
Constant	0.061 (0.258)	0.214 (0.229)	0.247 (0.248)	0.291 (0.228)
Observations	318	318	318	318
R^2^	0.337	0.482	0.398	0.496

Note: Standard errors are in parentheses

*, **, and *** indicate significance at the 10%, 5%, and 1% statistical levels, respectively.

Sources: The author compilating based on empirical results.

The estimation results of Regression (8) and (10) indicate that the expansion of BS can promote the use of agricultural machinery before and after controlling the FDAL. This suggests that the larger BS can reach the scale threshold for mechanical farming and enhance the incentive of farmers to use machinery. Similarly, the estimation results of Regression (9) and (10) show that the deepening of FDAL has a significant negative effect on the use of farm machinery under the condition of controlled and uncontrolled BS, which indicates that the reduction of arable land fragmentation level favors farmers’ machinery selection behavior.

In summary, off-farm employment, in general, promotes the machinery use behavior of farmers, thus reducing the intensity of CF application. Hypothesis H1 is confirmed.

Table 5 reports the results of model estimation for CF application intensity including interaction terms. The interaction term between off-farm employment and BS in Regression (11) has a significant negative effect on CF application intensity, indicating that the larger the BS becomes, the more farmers involved in off-farm employment tend to use machinery instead of labor, thereby reducing CF application intensity. If the BS is small, the reduction of labor factors will lead to factor substitution effect and off-farm employment will increase the intensity of CF application when farmers prefer to increase CF input to replace labor factor. The interaction term between off-farm employment and FDAL in Regression (12) has a significant positive effect on CF application intensity, indicating that the higher the FDAL becomes, the more farmers involved in off-farm employment tend to apply more CF as a substitute for the labor factor. The lower the FDAL becomes, the more farmers tend to use machinery instead of labor elements, so as to obtain the CF reduction effect of economies of scale and reduce CF application intensity. Hypothesis H2 and hypothesis H3 are confirmed.

**Table 5:** Model estimation results of CF application intensity of sample farmers (including interactive terms).

Variables	The predicted variable: ln(Cfertilizer)	
	Regression (11)	Regression (12)
Naemploy	-0.075*** (0.027)	-0.074*** (0.027)
Bscale	-0.025*** (0.001)	-0.025*** (0.001)
Nplots	0.006*** (0.001)	0.006*** (0.001)
Naemploy×Bscale	-0.012** (0.005)	-
Naemploy×Nplots	-	0.011*** (0.003)
The control variables	yes	yes
Regional dummy variables	yes	yes
Constant	7.146*** (0.044)	7.159*** (0.044)
Observations	318	
R2	0.809	0.814

Note: Standard errors are in parentheses

*, **, and *** indicate significance at the 10%, 5%, and 1% statistical levels, respectively.

Sources: The author compilating based on empirical results.

### 5.2 Instrumental variable estimation results

In this paper, a two-stage regression of the instrumental variables was conducted by using the 2SLS method and the estimation results for the instrumental variables are reported in Table 6. The results of the first stage estimation show that the village level off-farm employment network has a significant positive effect on the off-farm employment behavior of farm households. This shows that farmers can quickly access information and advice from acquaintances as well as help for employment outside the home through local off-farm networks. The higher the off-farm employment rate at the village level becomes, the greater the likelihood is that farm households will participate in off-farm employment. Farmers’ cropland topography has a significant positive effect on the BS and a significant negative effect on the FDAL. This suggests that the natural cut apart of rice BS and farmland plots by topographical factors makes the allocation of production factors by farmers regionally heterogeneity, that is, the use of machinery is more feasible in plain areas. Based on the empirical rule that the F-statistic of the first stage estimation should be greater than 10, the original hypothesis of "the existence of weak instrumental variables" is rejected. Hausman’s tests reject the original hypothesis that off-farm employment, scale of business, FDAL, and the corresponding interaction term are exogenous variables, indicating the existence of endogenous explanatory variables, and supporting the use of the instrumental variable method.

**Table 6:** Estimated results of instrumental variables.

Variables	Regression (13)	Regression (14)	Regression (15)	Regression (16)	Regression (17)
The first stage	Naemploy	Bscale	Nplots	Naemploy×Bscale	Naemploy×Nplots
VOEN	0.915*** (0.085)	-	-	-	-
Ttype	-	2.406*** (0.249)	-2.591*** (0.498)	-	-
VOEN×Ttype	-	-	-	7.662*** (0.525)	-6.120*** (1.030)
F-statistic	115.695	93.327	27.023	213.294	35.322
**The second stage**	**The predicted variable: ln(Cfertilizer)**				
Naemploy	0.052 (0.052)	-0.007 (0.037)	-0.005 (0.054)	-0.063** (0.027)	-0.054* (0.029)
Bscale	-0.026*** (0.001)	-0.046*** (0.004)	-0.011*** (0.004)	-0.025*** (0.001)	-0.026*** (0.001)
Nplots	0.007*** (0.001)	0.002 (0.001)	0.030*** (0.005)	0.007*** (0.001)	0.007*** (0.001)
Naemploy×Bscale	-	-	-	-0.025*** (0.007)	-
Naemploy×Nplots	-	-	-	-	0.031*** (0.009)
The control variables	yes	yes	yes	yes	yes
Regional dummy variables	yes	yes	yes	yes	yes
Constant	7.170*** (0.046)	7.133*** (0.058)	7.001*** (0.090)	7.140*** (0.044)	7.174*** (0.046)
Hausman Test	9.850***	74.000***	74.000***	5.630**	5.740**
Observations	318				
R^2^	0.789	0.651	0.272	0.804	0.783

Note: Standard errors are in parentheses

*, **, and *** indicate significance at the 10%, 5%, and 1% statistical levels, respectively.

Sources: The author compilating based on empirical results.

The results of the second stage estimation show that the effects of off-farm employment, scale of business, FDAL, and their corresponding interaction terms on CF application intensity are in the same direction as the benchmark model and generally consistent with the benchmark model at the significance level.

### 5.3 Robustness tests

The survey population includes 69 farm households that do not participate in off-farm employment. To confirm the robustness of the benchmark model results, the reduced sample size method is used to conduct robustness tests by excluding the sample data that do not participate in off-farm employment and selecting the remaining sub-samples as the new total sample. Table 7 reports the estimated results of the robustness test. The results show that the direction of impact and significance level of the sub-sample estimation results are consistent with the overall benchmark model after excluding the sample data of nonparticipating farmers employed off-farm, which verifies the robustness of the estimation results. Meanwhile, the instrumental variables estimation method can be used as a robustness test, which again verifies the robustness of the basic conclusions of the previous paper.

**Table 7:** Robustness test of the benchmark model.

Variables	The predicted variable: ln(Cfertilizer)					
	Regression (18)	Regression (19)	Regression (20)	Regression Test (21)	Regression Test (22)	Regression Test (23)
Naemploy	-0.634*** (0.067)	-0.322*** (0.054)	-0.269*** (0.048)	-0.490*** (0.059)	-0.229*** (0.043)	-0.266*** (0.049)
Bscale	-	-0.023*** (0.002)	-0.020*** (0.002)	-	-0.022*** (0.001)	-0.020*** (0.002)
Nplots	-	-	0.008*** (0.001)	0.012*** (0.001)	0.008*** (0.001)	0.008*** (0.001)
Naemploy×Bscale	-	-	-	-	-0.050*** (0.006)	-
Naemploy×Nplots	-	-	-	-	-	0.008 (0.011)
The control variables	yes	yes	yes	yes	yes	yes
Regional dummy variables	yes	yes	yes	yes	yes	yes
Constant	7.285*** (0.073)	7.224*** (0.054)	7.177*** (0.049)	7.205*** (0.064)	7.140*** (0.043)	7.179*** (0.049)
Observations	249	249	249	249	249	249
R^2^	0.574	0.769	0.818	0.687	0.858	0.819

Note: Standard errors are in parentheses

*, **, and *** indicate significance at the 10%, 5%, and 1% statistical levels, respectively.

Sources: The author compilating based on empirical results.

## 6. Discussion

### 6.1 Interpretation of results

Compared with the existing studies that mainly focus on the impact of off-farm employment on farmers’ overall CF application behavior and the difference of CF application intensity between regions, we found that off-farm employment has different effects on CF application intensity under different arable land resource endowments. Firstly, off-farm employment in general promotes the adoption of agricultural machinery, which achieves the economies of scale in rice planting and leads to a CF reduction effect. Affected by the small scale and scattered farmland plot, Chinese agricultural production modes are mainly represented by family management with small farmers as the main body. In recent years, the Chinese government has introduced corresponding measures to encourage farmers to transfer land. However, due to the limitation of arable land topography, land contract management right transfer is mainly concentrated in the north. Jilin Province, with a vast territory, is a main grain-producing area in China. Land transfer further expands the land scale, providing conditions for the popularization of machinery and the development of socialized service organizations. When the farmland plot scale is large, mechanical farming is a tool to improve the efficiency of resource utilization and achieve economies of scale. At this time, machinery is the optimal choice for factor reallocation by off-farm employment under the conditions of the large-scale farmland plot. Currently, although the fact that a large amount of small-scale and scattered farmland plot still exist, the arable land in Jilin Province shows a centralized trend. The improvement in farmland plot scale reduces the threshold for mechanical intervention, which provides an indirect pathway for off-farm employment to reduce the intensity of CF application.

Secondly, the difference in farm resource endowments regulates the relationship between off-farm employment and CF application. To compensate for the labor shortage effect caused by off-farm employment and stabilizing agricultural production, farmers tend to apply more CF on arable land with small BS and high fragmentation degree, which leads to the increase of CF application intensity That is because the smaller plot scale cannot meet the scale threshold for mechanical farming resulting in the inability of farmers to reconfigure the ideal production factors (machinery). Therefore, CF becomes the optimal choice for the allocation of production factor. Although the marginal return of successive increases in CF factor inputs is decreasing, the marginal return is positive. Meanwhile, the low price of CF factors makes the farmer increase CF input until the marginal return is zero.

Thirdly, off-farm employment provides opportunities for machinery selection, which significantly promotes farmers’ machinery adoption behavior as well as indirectly reduces CF application intensity in the reason of arable land with large BS and low fragmentation degree. As mentioned earlier, when the arable land endowment resource meets the threshold for mechanical farming, the optimal substitution factor for farmers is machinery. Farmers can improve resource utilization efficiency and obtain economies of scale through the round-tripping effect of mechanical farming and reduction technology, so as to reduce CF application intensity. This suggests that, unlike the case of direct substitution of CF, which is affected by the regulation effect, off-farm employment does not directly affect CF application in the large farmland scale scenario, but indirectly reduces CF application intensity by directly influencing the adoption of agricultural machinery.

### 6.2 Theoretical implications

This study enhances the current understanding of the role of off-farm employment in influencing CF application. Firstly, we discussed the differences in the role of off-farm employment on fertilizer application intensity in different arable land resource endowments. Existing micro studies mainly focus on the analysis of off-farm employment affecting the farmers’ overall CF application behavior from a single perspective [84–86]. The macro studies focus on the difference in CF application intensity caused by off-farm employment in different regions [11]. Both ignore that under the condition of different arable land resource endowments, off-farm employment leads to the change of production factor allocation, which in turn affects the difference of CF application. Therefore, we incorporate arable land resource endowment into the theoretical analysis framework to reveal two different mechanisms by which off-farm employment affects CF application. According to the logical clue of "off-farm employment—factor allocation—CF application", we explain it from a micro perspective. This study supplements the relevant literature on off-farm employment, factor allocation, and CF application, providing new insights on how to reduce and efficiently apply CF in the context of off-farm employment. Secondly, this study draws lessons from the new economics of labor mobility (NELM) theory to provide theoretical support for the proposed impact mechanism while expanding the theoretical boundaries of NELM based on CF application reduction and green production. Thirdly, this study reveals the regulatory effect of arable land resource endowment in the process of off-farm employment influencing CF application under different scenarios of BS and FDAL, providing empirical support. Fourthly, this study provides general policy implications for government administrators in most developing countries in the context of massive outflow of rural labor force.

### 6.3 Managerial implications

In addition to contributing to theory, our study provides some managerial insights for government administrators to seek to promote the reduction and efficiency of CF under the background of off-farm employment. Firstly, in recent years, the farmland in northeastern China has been gradually concentrated on specialized households, which not only improves the economies of scale of rice planting, but also has a positive impact on reducing CF application intensity. However, the arable land has not changed the essence of small-scale and fragmentation. Therefore, government administrators should actively guide farmers to participate in land replacement and integration in order to expand the BS as well as reduce the FDAL through continuous land transfer especially in flat areas, to obtain scale economy of CF reduction. Secondly, we point out that the use of machinery plays an important role in reducing the intensity of CF application. Limited by the specificity of agricultural machinery assets and high investment threshold, farmers generally have insufficient long-term investment motivation, but the contiguous planting of land transfer provides market capacity for agricultural machinery socialized service. As a result, agricultural machinery socialized service organizations come into being. However, the development of socialized service organizations depends on the contiguous planting of large-scale farmland plots and the low-cost purchase price of agricultural machinery. Therefore, the government should formulate different subsidy policies for agricultural machinery procurement according to the types and used times of agricultural machinery to improve the motivation of farmers and socialized service organizations. By increasing the round-tripping investment effect of farmers, we can obtain the economies of scale of mechanical farming and reduce the intensity of CF application. Thirdly, for small plots of arable land that cannot be transferred due to topographic restrictions, we should introduce social service organizations with accurate reduction technology, cultivate crops with high economic value and low CF demand and reduce the intensity of CF application through intensive cultivation.

### 6.4 Limitations and future research

Off-farm employment includes full-time off-farm employment and part-time off-farm employment. Part-time off-farm employment may be "off-farm work in leisure time and farm in busy time", which will not lead to the reduction of labor factors in agricultural production while causing the return of remittance effect. Due to the strong mobility of farm households involved in off-farm employment, it is difficult for us to obtain comprehensive information on part-time off-farm employment of farm households other than to extract data from the survey questionnaire. The estimation results of full-time off-farm employment and part-time off-farm employment will differ due to differences in household livelihood strategies. Therefore, an in-depth analysis is necessary to further investigate differences between the two different off-farm employment patterns affecting CF application.

In addition, this study uses cross-sectional data, which may lead to biased estimation results by ignoring differences in cropland quality across regions. Therefore, it is necessary to conduct a fixed-point tracking investigation to obtain panel data in the future to overcome the bias of model results.

## 7. Conclusions

Off-farm employment not only improves the livelihood of families in developing countries but also has a significant impact on agricultural production. In this paper, we analyzed the impact of off-farm employment on the mechanical use and CF input intensity of rice growers in Jilin Province, China. Descriptive statistical analysis and econometric results show that off-farm employment affects CF application intensity through two mechanisms. That is, off-farm employment affects CF application with situational dependence. Based on the viewpoint of factor allocation and NELM theory, this study proved the difference in the regulating effect of arable land resource endowment in the process of off-farm employment affecting CF application intensity. The results of the study show that the arable land resource endowment is mainly reflected in two aspects: the BS and the FDAL. There are differences in factor substitution behavior and CF reduction potential expressed by different arable land resource endowments. In general, off-farm employment increases farmers’ demand for mechanical farming, and the use of machinery is an effective means of reducing the intensity of CF application. If farmers have a small BS and dispersed farmland parcels, it can’t meet the scale economy of rice planting and even hinder the substitution of machinery for labor force. Therefore, the reduction effect of CF brought by off-farm employment may be limited and even increase the intensity of CF application. If farmers have a large BS and non-dispersed farmland parcels, the arable land endowment meets the scale threshold for mechanical farming to obtain the CF reduction effect of economies of scale. Therefore, off-farm employment will significantly contribute to CF reduction. Based on theoretical analysis, this study makes an empirical test conducted by using the sample data of 318 rice growers in Jilin Province. After overcoming the potential endogeneity in the econometric model, the robustness test based on sub samples also supports the above conclusions.

Scholars generally believe that off-farm employment is an important measure to improve family livelihood, and it will also have a significant impact on agricultural production. However, there is still controversy about how off-farm employment affects CF application. They argue that off-farm employment positively or negatively influences CF application or is influenced by differences in the timing of off-farm employment, type of off-farm employment, and crop species, resulting in differences in CF application. However, this paper holds that the fundamental reason why off-farm employment affects the application of CF lies in the difference in arable land resource endowment of farmers. After incorporating arable land resource endowment into the theoretical analysis framework and empirically analyzing the moderating role of arable land resource endowment, it is found that off-farm employment affects CF application through two mechanisms and produces different results. The findings of this study are not only applicable to understanding the impact of off-farm employment on machinery adoption and CF application, but can also enlighten how to influence inputs of other factors such as pesticides, herbicides, and water resources. At present, the confirmation of farmland property rights in China can accelerate the transfer and merger of land to a certain extent, thus contributing to the improvement of production efficiency and the reduction of CF application intensity. For the government, our findings provide a basis for decision-making on how to promote land management and reduce the intensity of CF application. Under the background of increasing off-farm employment, it affirmed the implementation direction of farmland confirmation and farmland transfer policy and the effectiveness of cultivating agricultural social service organizations. For farmers, when off-farm employment leads to labor shortage, increasing the BS and reducing the number of plots in the form of land transfer can reach the scale threshold of mechanical farming, and through the purchase of agricultural production services can obtain the CF reduction effect of large-scale operation and reduce the cost of CF inputs. Our research also shows that the scale of land can provide conditions for the use of modern equipment, reduce the input of agricultural chemicals, improve soil quality and agricultural non-point source pollution. Finally, it provides general policy suggestions for solving the agricultural non-point source pollution problem in developing countries with the background of the continuous outflow of the rural labor force.

Given the importance of off-farm employment in improving household livelihoods and increasing agricultural factor inputs, and the difference of the influence of cultivated land resource endowment on the intensity of CF application. The policy formulation should focus on reducing the restrictive factors in the process of land circulation concentration, especially in flat plain areas. For scattered plots that are severely restricted by topography, green organic agriculture should be encouraged to improve the ecological added value of products. In addition, due to the specificity of agricultural machinery assets and the high silent cost, the government should strengthen the support and cultivation of agricultural production service outsourcing organizations, give certain subsidies to agricultural machinery buyers, increase the scale effect of agricultural production and reduce the intensity of CF application through scale.

## Supporting information

S1 File (DO)

S1 Data (DTA)
